# Close relationship of a novel *Flavobacteriaceae* α-amylase with archaeal α-amylases and good potentials for industrial applications

**DOI:** 10.1186/1754-6834-7-18

**Published:** 2014-01-31

**Authors:** Chunfang Li, Miaofen Du, Bin Cheng, Lushan Wang, Xinqiang Liu, Cuiqing Ma, Chunyu Yang, Ping Xu

**Affiliations:** 1 State Key Laboratory of Microbial Technology, Shandong University, Jinan 250100, People’s Republic of China; 2 State Key Laboratory of Microbial Metabolism & School of Life Sciences and Biotechnology, Shanghai Jiao Tong University, Shanghai 200240, People’s Republic of China

**Keywords:** α-Amylases, Evolutionary position, Site-directed mutagenesis, Thermostability, Domain C

## Abstract

**Background:**

Bioethanol production from various starchy materials has received much attention in recent years. α-Amylases are key enzymes in the bioconversion process of starchy biomass to biofuels, food or other products. The properties of thermostability, pH stability, and Ca-independency are important in the development of such fermentation process.

**Results:**

A novel *Flavobacteriaceae Sinomicrobium* α-amylase (FSA) was identified and characterized from genomic analysis of a novel *Flavobacteriaceae* species. It is closely related with archaeal α-amylases in the GH13_7 subfamily, but is evolutionary distant with other bacterial α-amylases. Based on the conserved sequence alignment and homology modeling, with minor variation, the Zn^2+^- and Ca^2+^-binding sites of FSA were predicated to be the same as those of the archaeal thermophilic α-amylases. The recombinant α-amylase was highly expressed and biochemically characterized. It showed optimum activity at pH 6.0, high enzyme stability at pH 6.0 to 11.0, but weak thermostability. A disulfide bond was introduced by site-directed mutagenesis in domain C and resulted in the apparent improvement of the enzyme activity at high temperature and broad pH range. Moreover, about 50% of the enzyme activity was detected under 100°C condition, whereas no activity was observed for the wild type enzyme. Its thermostability was also enhanced to some extent, with the half-life time increasing from 25 to 55 minutes at 50°C. In addition, after the introduction of the disulfide bond, the protein became a Ca-independent enzyme.

**Conclusions:**

The improved stability of FSA suggested that the domain C contributes to the overall stability of the enzyme under extreme conditions. In addition, successfully directed modification and special evolutionary status of FSA imply its directional reconstruction potentials for bioethanol production, as well as for other industrial applications.

## Background

As large-scale substitution of petroleum-based fuels is needed for energy security and climate change considerations, bioethanol production from various starchy materials such as corn, wheat, cassava root, and starch have received much attention in recent years [1,2]. The starch-degrading enzyme α-amylases (EC 3.2.1.1) are the enzymes that hydrolyze starch, glycogen, and related polysaccharides by cleaving α-1,4-glucosidic linkages at random, and are regarded as the most important enzymes for bioethanol process, as well as a wide range of other industrial applications such as production of detergents, textiles, food, and paper [3]. Economically, it is desirable that the α-amylases used in starch liquefaction are active at high temperatures (approximately 90°C) [4]. To obtain ideal industrial enzymes, many comparative analyses and directed evolutions have been performed to elucidate the catalytic properties and stability parameters of glycosyl hydrolase family 13 (GH13) enzymes [5-7]. Moreover, many thermostable α-amylases were explored to meet the starch saccharification process. Thermostable α-amylases are produced by a wide variety of microorganisms, including thermophiles and mesophiles [8]. Among bacteria, *Bacillus* sp. is widely used for thermostable α-amylase production to meet industrial needs [9,10]. In addition, the most thermoactive α-amylases from hyperthermophilic archaea have attracted increasing attention and have been characterized from *Pyrococcus woesei*, *P. furiosus*, *Thermococcus profundus*, and *T. hydrothermalis*[11-14]. Some general strategies to increase the thermal stability of these enzymes have also been proposed and used for directed evolution, such as change of the secondary structure strengthening the hydrophobic interactions in intermolecular contacts, introduction of hydrogen bonds and salt bridges, and increase of the hydrophobicity of the protein surface [15,16].

Except for the α-amylase family 57, which comprises a few amylolytic enzymes from some hyperthermophiles [17,18], most of the α-amylases belong to GH13, based on the classification of Henrissat [19] and the Carbohydrate-Active Enzymes (CAZy) database at: http://www.cazy.org[20]. In this classification, GH13 is found to be the largest family of glycoside hydrolases, comprising the majority of the enzymes acting on starch, glycogen, and related oligo- and polysaccharides [21]. GH13 has been subdivided into 40 subfamilies on the basis of sequence similarity and phylogenetic reconstruction criteria [21,22]. Based on this division, the phylogeny of α-amylases is generally in agreement with their origin, such that most of the bacterial α-amylases are assigned to the GH13_5 subfamily, whereas GH13_6 includes α-amylases from plants, and the GH13_7 group comprises α-amylases of archaeal origin [21]. However, there are also some exceptions from such criteria, such as the close relationship between plant and archaeal α-amylases [8,23,24]. In addition, as inferred from the CAZy database, there is also another interesting exception for bacterial α-amylases: the hypothetic *Flavobacteriaceae* α-amylases belong to the GH13_7 subfamily, together with thermophilic archaeal α-amylases [20,25].

The family *Flavobacteriaceae* is one of the largest branches in the phylum *Bacteroidetes*. In this study, genomic analysis of a new species of this family revealed the presence of an open reading frame (ORF) encoding a novel α-amylase of *Flavobacteriaceae Sinomicrobium* (FSA). Since no such α-amylase was characterized and no evolutionary relationship was reported to date, in the present study, the evolutionary position, catalytic properties, and conserved regions of FSA were analyzed.

## Results

### Phylogenetic analysis

The phylogenetic analysis of the sequence of 16S rRNA gene revealed that strain 5DNS001 is a member of the family *Flavobacteriaceae* and exhibits the highest identity (96.3%) with *Sinomicrobium oceani*[26]. Based on the many differences with its relative strains, 5DNS001 would be classified as a novel species of the genus *Sinomicrobium* and designated as *Sinomicrobium* sp. 5DNS001.

**Figure 1:**
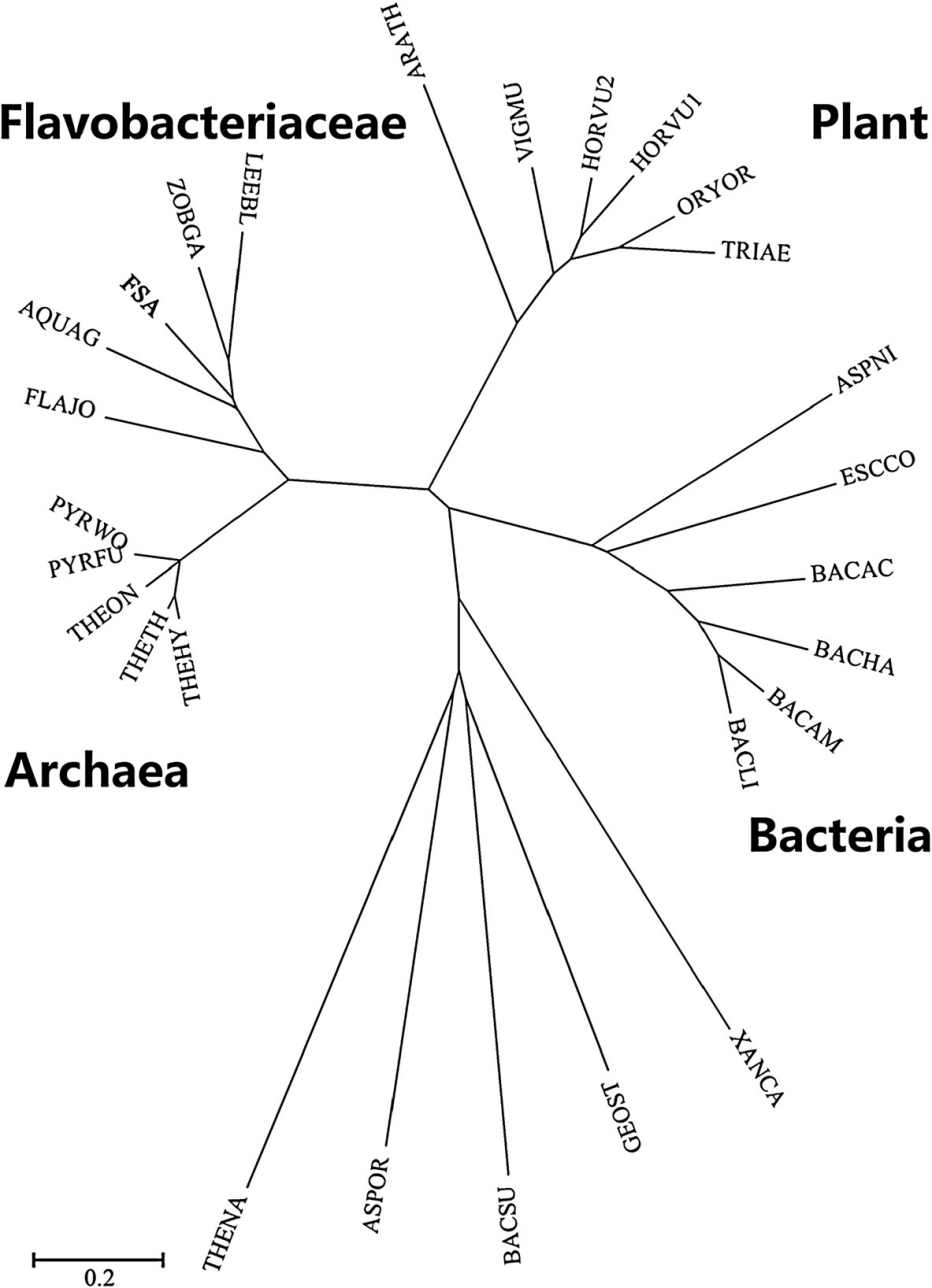
**Phylogenetic tree of FSA and its closest relatives.** FSA, *Flavobacteriaceae Sinomicrobium* α-amylase, present study; LEEBL, *Leeuwenhoekiella blandensis*; ZOBGA, *Zobellia galactanivorans*; FLAJO, *Flavobacterium johnsoniae*; AQUAG, *Aquimarina agarilytica*; PYRFU, *Pyrococcus furiosus*; PYRWO, *P. woesei*; THEHY, *Thermococcus hydrothermalis*; THEON, *T. onnurineus*; THETH, *T. thioreducens*; ARATH, *Arabidopsis thaliana*; VIGMU, *Vigna mungo* seed; ORYOR, *Oryzeae Oryza*; TRIAE, *Triticum aestivum*; HORVU1, *Hordeum vulgare*; HORVU2, *H. vulgare*; ASPOR, *Aspergillus oryzae*; ASPNI, *A. niger*; THENA, *Thermotoga naphthophila*; ESCOO, *Escherichia coli*; GEOST, *Geobacillus stearothermophilus*; BACLI, *Bacillus licheniformis*; BACSU, *B. subtilis*; BACAC, *B. acidicola*; BACHA, *B. halmapalus*; BACAM, *B. amyloliquefaciens*; XANCA, *Xanthomonas campestris*. Bootstrap percentages >50% (based on 1,000 replications) are shown at branch points.

Based on the annotation results of the genomic information of 5DNS001, a 1437-bp gene encoding a novel α-amylase exhibits the highest identity (65.1%) with the putative α-amylase of the *Flavobacteriaceae* species *Zobellia galactanivorans*, and 50.8% identity with the putative α-amylase of the *Flavobacteriaceae* species *Flavobacterium johnsoniae (FLAJO)*. Moreover, in comparison with other identified proteins, FSA exhibits the highest identity of 43.8% with the α-amylase of *P. woesei (PWA).* However, it shows lower than 30% identity with the α-amylases from other bacteria or from the plant. The deduced amino acid sequence of the α-amylase coding region includes a putative signal peptide of 26 amino acids and the mature enzyme of 452 amino acids. As shown in Figure 1, in comparison with plant, fungal, and other bacterial amylases, amylases from thermophilic archaea and *Flavobacteriaceae* clearly showed the nearest evolutionary relatedness when placed on adjacent branches of a larger common cluster. The *Flavobacteriaceae* α-amylases were clustered together with hyperthermophilic archaeal α-amylases of the family *Thermococcaceae*. In addition, the plant α-amylases were closest to this cluster whereas other origins from fungi and bacteria were located at further distances.

### Multiple alignments

**Figure 2:**
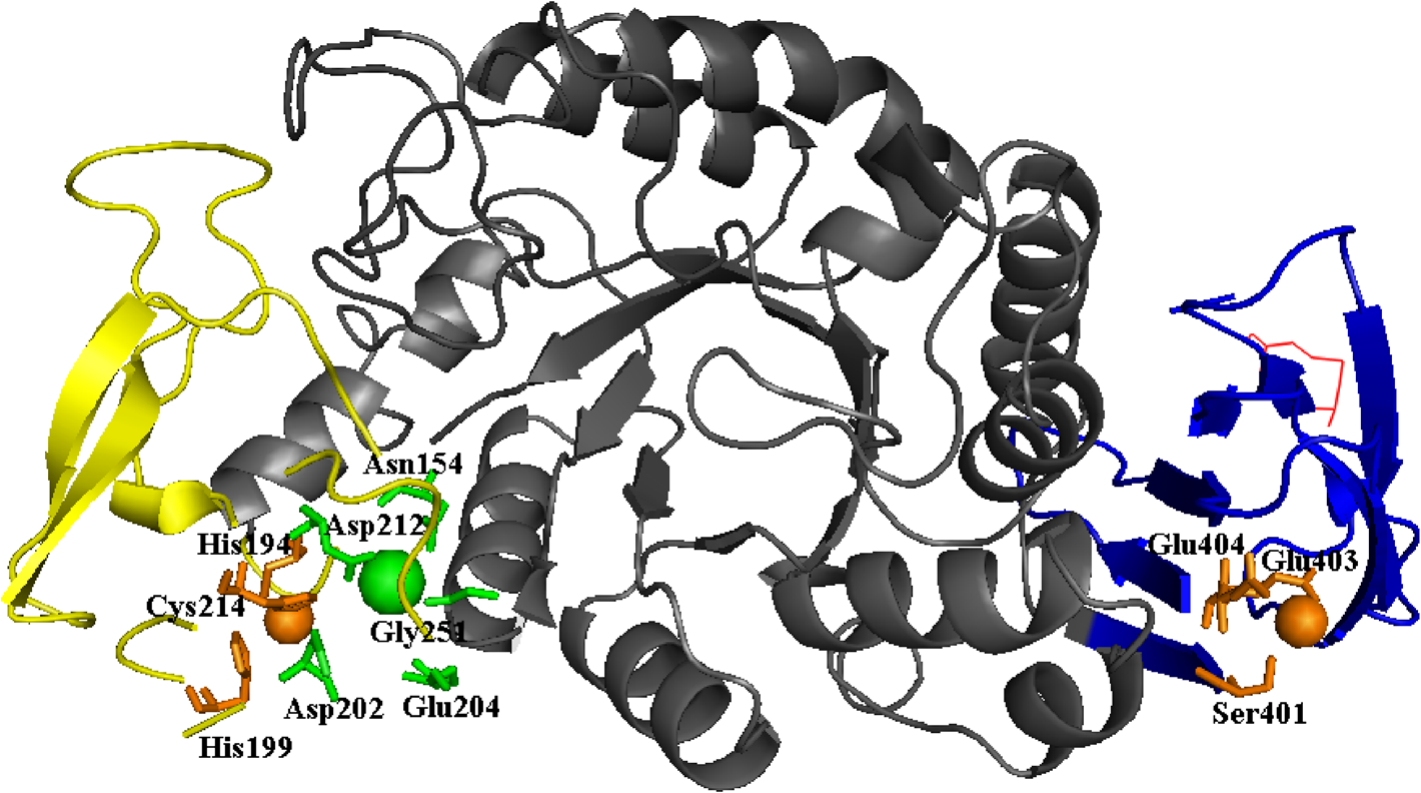
**Homology model of FSA and its putative metal-binding sites.** The Ca^2+^ and Ca^2+^-binding residues are marked as green; the Zn^2+^ and Zn^2+^-binding residues are marked as orange; and the introduced disulfide bond is marked as a red line. Domain A is represented as grey; domain B is represented as yellow; and domain C is represented as blue. FSA, *Flavobacteriaceae Sinomicrobium* α-amylase.

**Figure 3:**
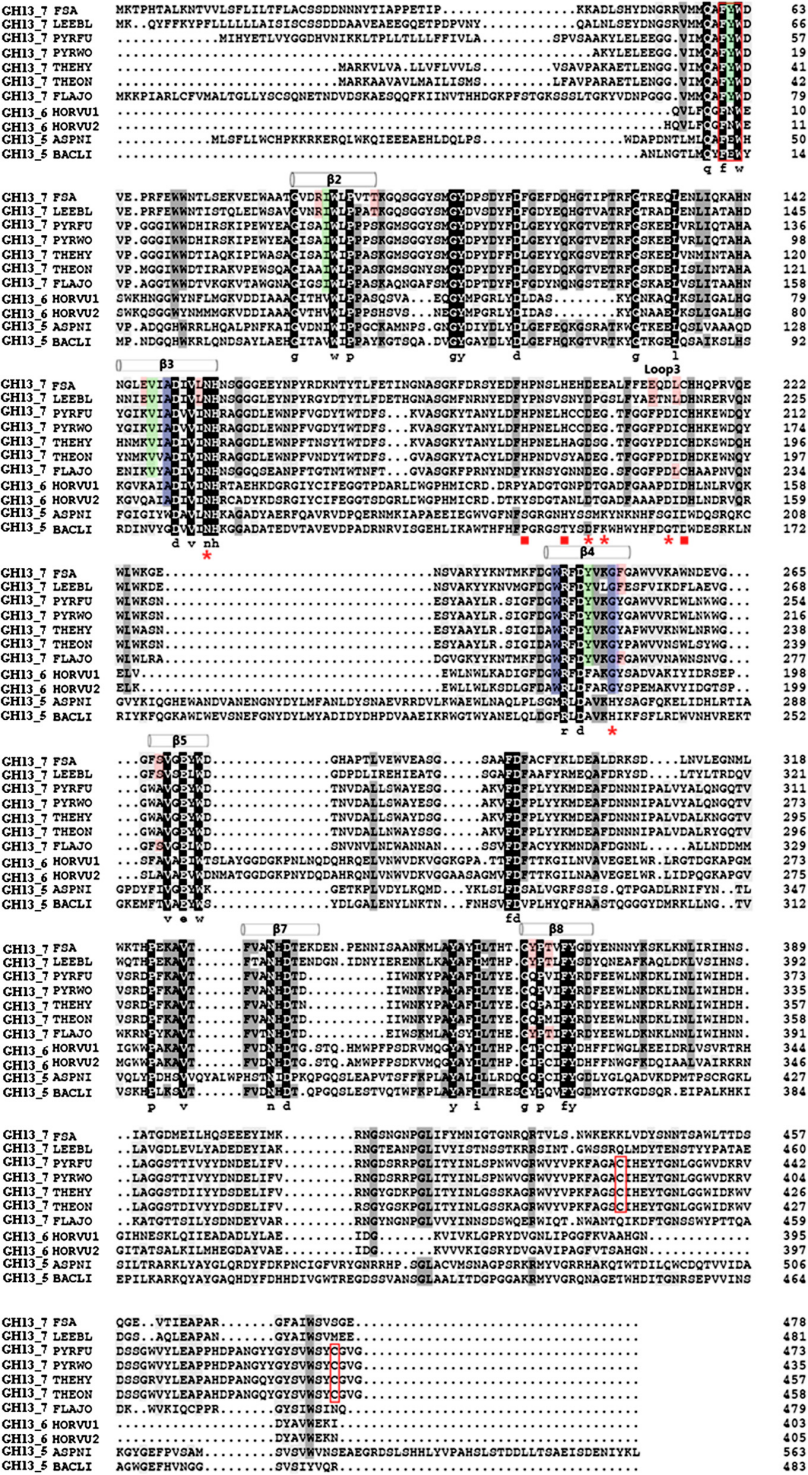
**Multiple sequence alignment of FSA and other GH13_5, GH13_6, and GH13_7 α-amylases.** Dark and grey backgrounds were adopted to highlight the positions of identical and similar residues, respectively. The positions of the amino acids coordinating the zinc ion are marked as ■; those coordinating calcium are marked as *; the mutated residues and FYW region are marked as a red box; the residues conserved in GH13_7 sequences are marked as green; residues conserved in *Flavobacteriaceae* amylases are marked as red; and residues conserved in both GH13_6 and GH13_7 sequences are marked as blue. FSA, *Flavobacteriaceae Sinomicrobium* α-amylase; GH13, glycosyl hydrolase family 13.

As predicted by PSIPRED and based on the homology model of Figure 2, FSA contains three distinct domains (A, B, and C) and shares outstanding similarity to other α-amylases. Domain A, containing residues 1 to 155 and 216 to 393, folds into an (α/β)_8_-barrel. As shown in Figure 3 of multiple sequence alignments, most of the highly conserved residues are located at domain A, especially at the six conserved regions of strands β2, β3, β4, β5, β7, and β8. Besides the three established active site residues of Asp247 in β4, Glu272 in β5, and Asp334 in β7 (unless otherwise specified, all amino acid numbering correspond to FSA), several residues are well conserved along all three subfamilies of GH13_5, GH13_6, and GH13_7: Gly85, Trp90, Pro92, Val152, Asn154, His155, Arg245, Asp247, Val269, Trp273, Asn332, Gly363, Pro364, Tyr368, and Phe377. Some of these conserved residues locate in the active center and interact with a number of highly conserved connections (that is, β4, β5, and β7 of the (α/β)_8_-barrel), such as the carboxyl groups of Trp273, Arg245, and Phe292 form hydrogen bonds with acarbose and predicted to be substrate-binding sites [15]. In addition, the conserved His155 locating near the Ca^2+^ serves as a Ca^2+^-binding site [15]. As shown in Figure 3, in addition, some residues are shared by both the GH13_5 and GH13_6 subfamilies (that is, Leu91, Ala149, Trp244, and Gly251) and others are shared only by the GH13_5 subfamily (that is, Ile89, Val147, and Tyr258). Furthermore, some residues are exclusive of the *Flavobacteriaceae* α-amylases: Arg88, Thr95, Glu146, Leu153, Glu210, Leu213, Phe252, Ser268, Tyr363, and Thr365. In addition, Ala331 in the i - 3 position from the transition state stabilizer Asp334 is only conserved in GH13_5.

Domain B of FSA (residues 156 to 216) inserted between the secondary structures α3 and β3 is one of the smallest α-amylase B domains. It is constituted of short β**-**sheets (three to four residues). As indicated from multiple alignments, BACLI and ASPNI have the longest B domains consisting of up to 100 residues, whereas the four thermophilic archaeal α-amylases together with FLAJO have the shortest regions consisting of 58 residues [15]. The remaining two *Flavobacteriaceae* α-amylases have the same size of 61 residues with the two plants of α-amylases. The five residues involved in Ca^2+^ binding (Asn110, Asp155, Gly157, Asp164, and Gly202: PWA number) in PWA are conserved in *Flavobacteriaceae*, archaeons, and plants, with the exception of Gly157 in PWA that is replaced by Glu in FSA (Glu202). In addition, the three Zn^2+^-binding residues in PWA (His147, His152, and Cys166) are all conserved in FSA (His194, His199, and Cys214) and the other three archaeal sequences, but no conservation was found in the other two sequences from *Flavobacteriaceae*.

Domain C in the C-terminal portion of the protein (residues 394 to 477) folds into a Greek key motif and contains the same numbers of residues as those of the two *Flavobacteriaceae* α-amylases, whereas the four thermophilic archaeal α-amylases contain nine additional residues. Among the five cysteines (Cys153, Cys154, Cys166, Cys388, and Cys432) in PWA, only Cys166 (Cys214 in FSA) is conserved in the FSA and FLAJO sequences. Moreover, an additional cysteine at position 296 (FSA numbering) is present in the FSA and FLAJO sequences. As revealed by the crystal structure of PWA, the two disulfide bonds present in the protein structure are formed by two adjacent cysteines (Cys153 and Cys154) in domain B and by Cys388 and Cys432 in domain C, respectively. However, as conferred from the homology model shown in Figure 2, no disulfide bonds were identified in FSA. The additional Zn^2+^-binding site observed in domain C of PWA (Asp347, Asp349, and Glu350) is well conserved among the *Flavobacteriaceae* and archaeon sequences, with the exception of FSA in which the corresponding residues are Ser401, Glu403, and Glu404 (Figures 2 and 3).

### Physical properties of the recombinant protein

**Figure 4:**
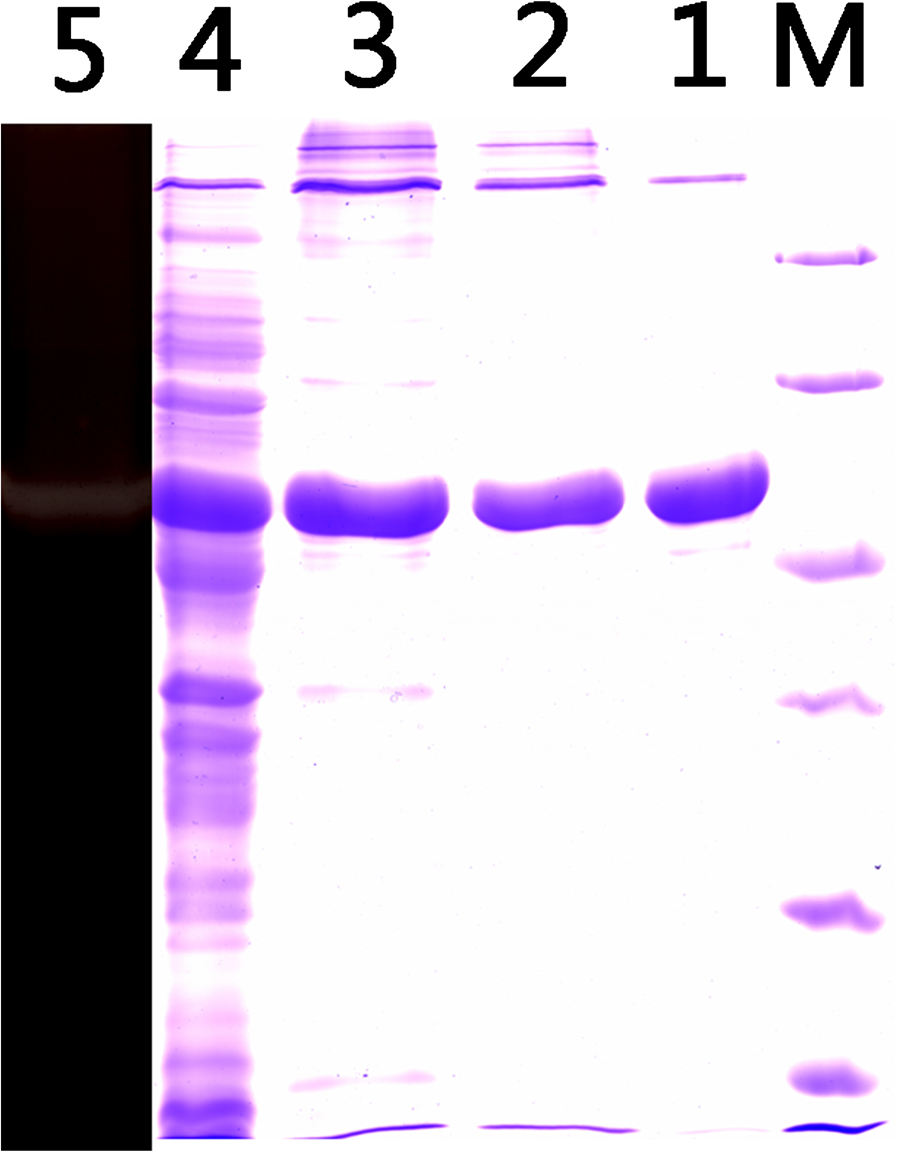
**SDS-PAGE and starch-containing PAGE analyses of FSA.** Lane M, marker; lane 1, culture supernatant of the induced transformant harboring FSA; lane 2, eluted protein after the first HisTrap affinity column; lane 3, eluted protein after the second HisTrap affinity column; lane 4, eluted protein after Superdex 200 column; and lane 5, FSA in starch-containing PAGE. FSA, *Flavobacteriaceae Sinomicrobium* α-amylase.

In the final plasmid pETDuet/FSA, the amylolytic activity was detected on Luria-Bertani (LB) agar plates according to the method described by Jørgensen *et al*. [27]. After a three-step purification procedure, the purified protein displayed a clear protein band of 52 kDa on SDS-PAGE and one clear band of amylolytic activity, and had a specific activity of 328.6 U·mg^-1^ at 50°C (Figure 4).

**Figure 5:**
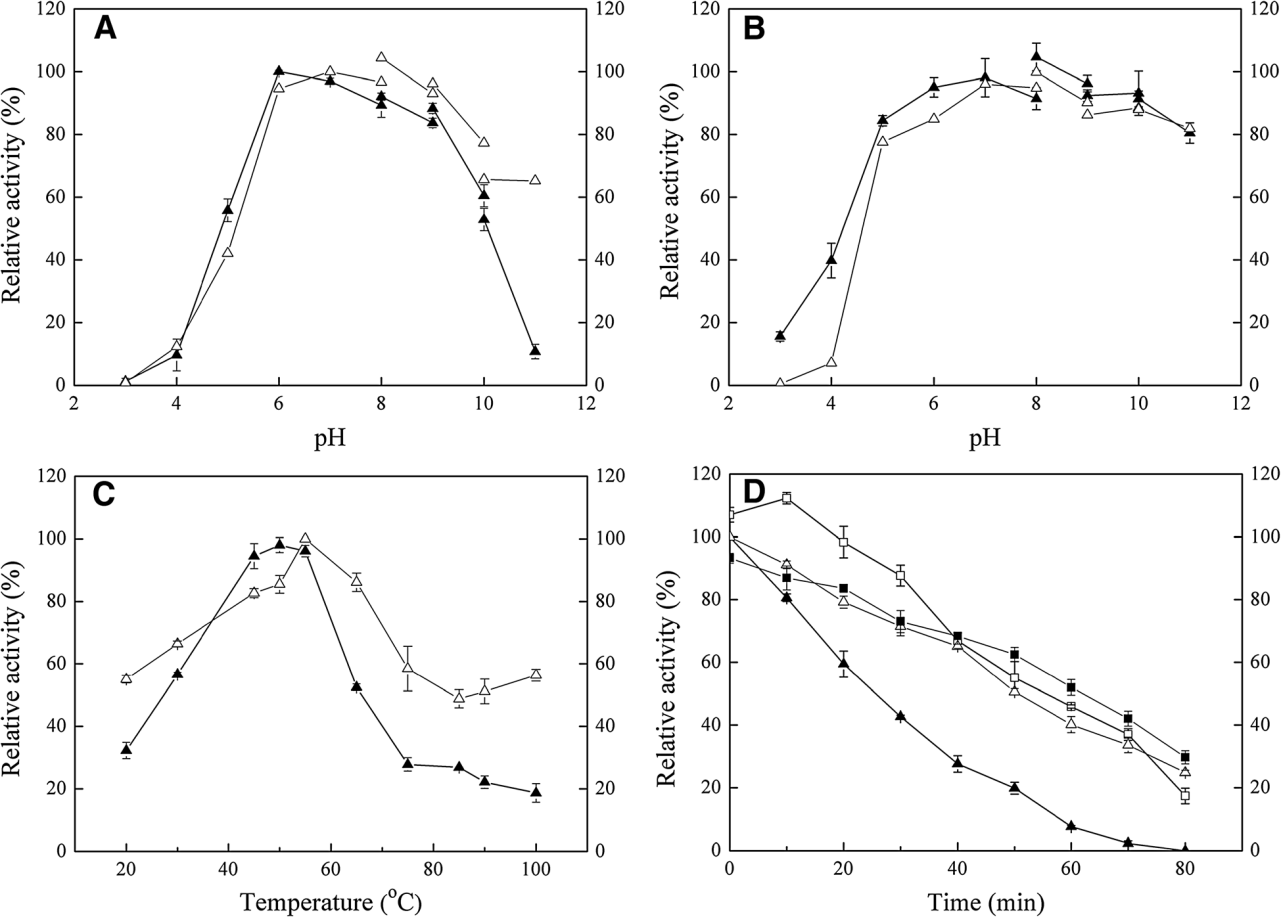
**Effects of pH and temperature on the activity of FSA and mutated protein FSAΔSK. (A)** pH profile; **(B)** stability of enzymes at various pH values; **(C)** temperature profiles; and **(D)** time-course curves of enzyme activities at 50°C. ▲ represents the relative activities of FSA at investigated conditions; and ∆ represents the relative activities of FSAΔSK at investigated conditions. In **(D)**, ▲ represents the relative activity of FSA at 50°C with no Ca^2+^ addition; ∆ represents the relative activity of FSAΔSK at 50°C with no Ca^2+^ addition; □ represents the relative activity of FSA at 50°C in the presence of 1 mM Ca^2+^; and ■ represents the relative activity of FSAΔSK at 50°C in the presence of 1 mM Ca^2+^.

Figure 5A shows the pH profiles for FSA activity. The enzyme displayed high activity at pH 6.0 to 9.0, with maximum activity at pH 6.0. Moreover, the enzyme is much more stable in the pH range of 6.0 to 11.0, with no loss of activity after incubation for up to 3 hours. However, it retained lower activity and stability when the pH was lower than 4.0 (Figure 5B).

The optimal temperature for FSA was determined at pH 6.0 by measuring the activity across a temperature range from 20°C to 80°C. As shown in Figure 5C, the enzyme has maximum α-amylase activity at 50°C and it retains more than 50% activity at 60°C. The enzyme is unstable at high temperature, with a half-life of approximately 25 minutes at 50°C. Different Ca^2+^ concentrations differently influence the enzyme thermostability (Figure 5D). Ca^2+^ could obviously improve the enzyme thermostability but the improvement declined as the Ca^2+^ concentration increased from 1 mM to 5 mM (data not shown). Therefore, 1 mM Ca^2+^ was selected as the proper dose for thermostability measurements. Consequently, as shown in Figure 5D, we determined an increased half-life of FSA of 55 minutes at 50°C.

The purified enzyme hydrolyzes soluble starch, amylopectin, and dextrin as shown in Table 1, whereas no activity was observed toward substrates of maltose, dextran, xylan, and β-cyclodextrin. The influence of various metal ions on the activity of FSA was tested at pH 6.0 and 50°C by measuring the enzyme activity in the presence of 5 mM metal ions (Table 2). The activity was strongly inhibited by the presence of Zn^2+^, Cu^2+^, Co^2+^, Mn^2+^, Ni^2+^, and Cd^3+^, whereas the addition of Ca^2+^ and K^+^ resulted in little increase of activity (12% and 13%, respectively). The kinetic parameters *V*_max_ 0.182 mg (ml·min)^-1^, *K*_m_ 5.361 mg ml^-1^, and *K*_cat_ 5.93 s^-1^ of FSA were calculated from the measured activity using a Lineweaver-Burk plot.

**Table 1:** **Substrate specificity of the α-amylase FSA from *Sinomicrobium *** **sp. 5DNS001**

**Substrate**	**Relative activity (%)**
Soluble starch	100.0
Amylopectin	66.7
Dextrin	38.8
Maltose	0
Dextran	0
Xylan (Birchwood)	0
Beta-Cyclodextrin	0

**Table 2:** The influence of different metal ions on the activity of FSA

**Metal ion**	**Relative activity (%)**
K^+^	113.2
Ca^2+^	112.5
Ba^2+^	53.1
Mg^2+^	52.3
Ni^2+^	10.4
Co^2+^	7.0
Cd^3+^	0
Mn^2+^	0
Cu^2+^	0
Zn^2+^	0
EDTA	0

### Properties of the mutant protein

The plasmid of FSAΔSK carrying the mutated gene was obtained by replacing S450 and K415 with cysteines. As shown in Figure 5A, after purification, the mutated protein presented a much broader pH range of activity than that of wild type FSA. The activity at pH 11.0 is much higher than that of the wild type protein (60% relative activity in FSAΔSK, and only 10% relative activity observed in wild type FSA). Similarly, the mutated protein also showed higher stability in neutral or alkaline environments (Figure 5B). Importantly, the thermal properties of the mutant enzyme presented a significant improvement deriving from the establishment of the disulfide bond in domain C. As shown in Figure 5C, FSAΔSK was active over a broad temperature range (20°C to 100°C) and had an optimum activity at 55°C. In contrast to the inactivity of the wild type enzyme at 100°C, more than 50% activity was detected at 100°C for the mutated protein. Despite the absence of Ca^2+^, FSAΔSK exhibits an obviously better thermostability than that of the wild type enzyme. The wild type enzyme exhibited a time-dependent decrease in amylolytic activity and had a half-life of 25 minutes at 50°C, whereas the half-life for FSAΔSK was increased to 55 minutes under the same temperature. It is interesting to note that no obvious improvement of the thermostability of the mutated enzyme was detected with the addition of 1 mM Ca^2+^. As shown in Figure 5D, nearly similar curves were observed in the presence or absence of Ca^2+^. In the first 10 minutes of the measurement, a slight inhibition of the thermostability was observed followed by a little improvement. Such Ca-independent thermal stability is different from that of the wild type α-amylase, as well as many reported α-amylases.

## Discussions

Despite the low identity (<10%) between the main groups of organisms, most of the within-kingdom α-amylases shared higher similarities. Based on the global phylogenetic tree deriving from various α-amylases of different origins [28], most of the kingdoms developed their own branches in the tree. However, some exceptions exist concerning bacteria. Obviously, some bacterial, but animal-, plant-, and fungi-like α-amylases scattered in related clusters. In the present study, the relatively small phylogenetic analyses among different sources of GH13_5, GH13_6, and GH13_7 α-amylases are in agreement with the conclusions from Janeček, according to whom plant and archaeal α-amylases from the family GH13 are sequentially similar and evolutionarily related [8]. Bacterial FSA and their *Flavobacteriaceae* analogues have a special distribution. They share a large branch with thermophilic archaeal α-amylases. Such an evolutionary relationship could be explained by the subfamily divisions of the GH13 family according to the CAZy database. Based on the division, numerous bacterial α-amylases are grouped into the GH13_5 subfamily except seven *Flavobacteriaceae* sequences. These seven exceptions belong to the GH13_7 subfamily along with 17 archaeal α-amylase sequences. Among these seven α-amylases, the enzyme from *F. johnsoniae* was the closest to the archaeal enzymes. Such a close relationship was also confirmed by the multiple alignments, in which numerous conserved residues were shared between FLAJO and archaeal α-amylases.

FSA possesses the common characteristics of other GH13 α-amylases: it adopts an (α/β)_8_-barrel supersecondary structure with four to seven conserved regions and three consensus residues (that is, Asp247, Glu272, and Asp334). Besides these characteristics, there are also some features shared with other α-amylases or specific to FSA α-amylase.

### Domain A

Obviously, most of the conserved residues were located in the six conserved β-sheets of domain A. Structural information and site-directed mutagenesis of α-amylases suggested that some of these residues are important for substrate recognition, specificity, binding, and enzyme stability. Among these highly conserved residues, it is well established that His155 plays an essential role in the binding of the substrate [29-31]. In addition, the carboxylate groups of Trp273, Arg245, and Phe292 form hydrogen bonds with acarbose and are predicated to be substrate-binding sites. Besides these sites, a short conserved stretch covering the strand β1 of the catalytic (α/β)_8_-barrel was found in various α-amylases. Generally, the conserved groups are FYW in archaeal, FNW in plant, and FEW in animal α-amylases [32]. As shown in Figure 3, the three *Flavobacteriaceae* sequences also belong to the FYW group. In this group, Tyr39 in *T. hydrothermalis* was found to contribute to thermostability [33]. Additional conserved sites were found in GH13_6 and GH13_7 subfamilies. Janeček demonstrated that these two families are closely evolutionary related [8]. In particular, Gly251 in the position of i + 4 from the catalytic nucleophile Asp247 is a Ca^2+^-binding site and is only found in these two subfamilies.

### Domain B

It is proposed that domain B, which is inserted within the (α/β)_8_-barrel at the β3 and α3 connection, is the most variable region in the α-amylase family [34,35]. However, as shown in Figure 3, high similarity was observed among archaeal and *Flavobacteriaceae* α-amylases with many residues conserved. It was demonstrated by continuous site-directed mutagenesis that domain B plays an important role in liquefying α-amylases, because its rigidity offers a substantial improvement in thermostability in *B. licheniformis* α-amylase (BLA) compared with *B. amyloliquefaciens* α-amylase [5,36]. With few exceptions, all the known α-amylases contain a conserved Ca^2+^ ion located at the interface between domain A and B which is essential for their catalytic activity [4,15,35]. The role of the conserved Ca^2+^ is mainly to retain the structural rigidity and activity of the α-amylases [37,38]. Some Ca-independent enzymes have also been reported, such as the thermostable α-amylases from *B. thermooleovorans* NP54 and *P. furiosus*[39,40], and few *Bacillus* α-amylases [16,41]. In FSA, Ca^2+^ did not influence its activity, with only about 10% increase in the presence of 5 mM Ca^2+^. However, the thermal stability of FSA was effectively enhanced by the addition of Ca^2+^, with the half-life of FSA increased by about two fold at 50°C. Therefore, differently from PWA, which does not require Ca^2+^ for its thermostability, the Ca^2+^ ion plays an important role in enzyme thermostability as a stabilizer, yet not as an activator. However, as inferred from the homology model and sequence alignment, the Ca^2+^-binding sites are also predicted in FSA, with the replacement of only one residue at these binding sites. As shown in Figure 2, the whole binding site appears looser than that of PWA. This might be an explanation for the differences of the two enzymes, both in the Ca^2+^ ion requirements and the thermostability levels. In accordance with the novel two-metal center (Ca, Zn) in the PWA structure, a closely similar binding center was also predicted. The Zn^2+^-binding site with three conserved residues, including Cys178 (Cys166 in PWA), is also found in FSA. It has been demonstrated that Cys166 was not involved in disulfide bridge formation, while it served as one of the coordinating ligands of the PWA (Ca, Zn) metal binding site. If the zinc site of this two-metal center is abolished by replacing its cysteine ligand (Cys166), the thermostability is drastically reduced [42]. Mutagenetic analysis also indicated that the recovery of the Zn^2+^-binding residues (His175 and Cys189) enhanced the thermostability of α-amylase in *T. onnurineus* NA1 [43].

By sequence comparison and use of Cys166 as an indicator, Linden *et al*. [15] have demonstrated that the Zn^2+^ site of the two-metal center is present only in *P. woesei* and its close homolog *T. hydrothermalis* (84% sequence identity). Even in the more closely related Archaea sequences from *P. kodakaraensis* and *Thermococcus* sp., this cysteine is replaced by an alanine. However, it is interesting to note that such residue is conserved in FSA, an enzyme relatively far from PWA compared to the α-amylase from *P. kodakaraensis* and *Thermococcus* sp. in phylogenetic relationships. However, no such site was found in another two *Flavobacteriaceae* sequences.

### Domain C

This all-β domain is also variable in dimensions and its functional role has not been completely recognized [5]. Domain C in some plant *Amy1* and archaeal PWA was found as the substrate-binding site [15,44]. In addition, some truncation experiments of domain C suggested that the enzyme activity, as well as stability, was not influenced by the removal of part of the domain [45,46]. However, with regard to the structure of isoamylase from *Pseudomonas amyloderamosa*, this domain was supposed to contribute to the overall catalytic domain stability by shielding the hydrophobic residues of the barrel [47]. It has been demonstrated that the C terminus of *Lactobacillus amylovorus* α-amylase plays a positive role in the thermostability of the enzyme [48]. The structure of PWA displays an additional metal-binding site (Zn^2+^ or Mg^2+^) in the loop connecting β-strands 1 and 2 of domain C. It has been predicted that this metal binding site has a stabilization role, which may be further enhanced by a nearby disulfide bridge connecting Cys388 and Cys432 [15]. In the present study, the successful introduction of a disulfide bond at these two sites resulted in significant improvement of the activity and thermostability of FSA. The activity temperature range of FSA was increased to a large extent, with about 50% activity detected at 100°C. Moreover, in comparison with the wild type protein, the half-life of the mutated protein at 50°C increased by about two fold. As for the activity at different pH conditions, it is worth noting that the pH profile of the mutated protein was also shifted to higher values. Many known α-amylases including BLA contain structurally essential Ca^2+^ to maintain their activity and thermostability. However, these Ca^2+^ are often removed by chelating agents such as zeolite and EDTA, and in turn result in the inactivity of enzymes [49,50]. Therefore, Ca-independent enzymes are preferable than Ca-dependent enzymes for industrial applications. In comparison with other *Bacillus* α-amylases (for example BLA), it not only shows higher activity over a broad pH range from 6.0 to 11.0, but also does not require Ca^2+^ for its structural stability. When compared with those thermophilic α-amylases of archaea origin (for example PWA), Ca^2+^ is not necessary for its activity and stability. However, the requirements of anaerobic and extreme growth conditions make them difficult to obtain a sufficient amount of these cells for large-scale enzyme production. Meanwhile, the heterologous proteins were often deposited within the host cells in the form of insoluble inclusion bodies which makes high heterologous expression difficult [51]. In the present study, good properties of broad pH profile, good pH stability, Ca-independent, and high expression in host, in combination with special evolutionary status implied good potentials of FSA for various applications.

These improvements demonstrated the important stability role of the disulfide bond in FSA under extreme environments. As shown in Figure 2, a corresponding Zn^2+^ was also predicated in domain C corresponding to the conserved residue of E404 in FSA. As shown in Figure 3, this residue is only conserved in the four archaeal and three *Flavobacteriaceae* sequences. Therefore, as predicated for PWA, the existence of a disulfide bond near the zinc site would play a stability role for the enzyme. Consequently, domain C is supposed to be important in retaining the protein structure under extreme conditions.

## Conclusions

In summary, the novel *Flavobacteriaceae* α-amylases in this study exhibited special evolutionary status and some similar properties such as novel (Ca, Zn) two-metal center with thermophilic archaeal α-amylases. The special evolutionary position suggests its good potentials for thermostability mechanism investigation and directional reconstruction potentials for bioethanol production. Based on the sequence comparisons and homology model analysis, a single disulfide bond in domain C was introduced in the enzyme and resulted in a significant improvement of the enzyme’s properties, including pH profile shifted toward alkaline pH, thermal activity and thermostability increased to a large extent, and Ca^2+^ requirement for thermostability disappeared. This successful introduction demonstrates the important role of domain C in retaining protein stability under extreme conditions. Improved catalytic properties of the engineered enzyme imply good potentials for starch slurry in the majority of industrial production of detergents, textiles, food, paper, and bioethanol.

## Methods

### Sampling and strain isolation

Soils were collected from a salt-making ruin in Dongying city in China, where the Yellow River flows into the sea (2 m, N37°14′50″ E118°41′11″). The salinity of the soil sample is 0.6% and the pH is 9.3. The strain was isolated from an enrichment of the soil by using the following enrichment medium (1 L): 20 g of MgCl_2_·6H_2_O, 5 g of K_2_SO_4_, 0.1 g of CaCl_2_, 0.1 g of yeast extract, 0.5 g of NH_4_Cl, 0.05 g of KH_2_PO_4_, 50 g of NaCl, 0.2 g of Tryptone, 0.5 g of casein, and 2 g of citrate sodium. The medium pH was adjusted to 9.0 with phosphate buffer. After incubation at 25°C for 1 week, the strain 5DNS001 was obtained by spreading the enrichment on the plate, followed by purification.

After cultivation at 25°C in LB medium, the genomic DNA of the strain was isolated with the DNA extraction Kit (Promega, Fitchburg, WI, USA). The 16S rRNA gene was amplified using the universal primers 27f and 1492r according to a previous report [52], ligated into the pGM-18 T vector (Promega), and transformed into *Escherichia coli* DH5α competent cells for sequencing.

### Sequence multiple alignment and homology modeling of the FSA

Genome sequencing for strain 5DNS001 was performed at the Beijing Genomics Institute (BGI, Beijing, China). A genomic DNA library with insert sizes of 500 bp was constructed and sequenced following the Solexa sequencing protocols (Illumina, Inc, San Diego, CA, USA). The sequence of the 5DNS001 gene was submitted to Prokaryotic Genome Automatic Annotation Pipeline (PGAAP) via the website: http://www.ncbi.nlm.nih.gov/genomes/static/Pipeline.html. Comparatively, a second annotation of rapid annotations using subsystems technology (RAST) service was used to identify and categorize the features of the genome [53]. One ORF designated as *FSA* putatively encoding the α-amylase FSA was selected for further study [GenBank:KC441955].

The amino acid sequence of FSA as well as that of other α-amylases obtained from the CAZy database, including GH13_5 (bacteria *B. licheniformis*; fungi *Aspergillus niger*), GH13_6 (plant *Hordeum vulgare*), GH13_7 (thermophilic archaeon *P. woesei*, *P. furiosus*, *T. hydrothermalis*, and *T. onnurineus*; *Leeuwenhoekiella blandensis* and *F. johnsoniae*), and other bacterial, fungal, and plant α-amylases, were selected for multiple alignments and phylogenetic analyses. Multiple alignments were conducted using the Clustal_X program [54], and the phylogenetic analyses were carried out with MEGA 4 [55]. The homology model of FSA was built using the automated SWISS-MODEL server, by using the crystal structure of α-amylase PWA [PDB:1MWO] from *P. woesei* as the template [56,57]. The sequence identity between the template and FSA was 43.8%. Visualization and analysis of the structures were undertaken using PyMOL (http://www.pymol.org).

### Overexpression and purification of the wild type protein FSA

Based on the sequence of FSA, the PCR product was generated by using the primers: A-f (5′-CG*GGATCC*GGATGACAATAATAATTATAC-3′) and A-r (5′- GAC*GTCGAC*TTACTCTCCCGAAACC-3′), where the restriction sites *Bam*HI and *Sal*I are **italicized**, respectively. The PCR product was digested, ligated into the expression vector pETDuet-1, and transformed into *E. coli* BL21-CodonPlus. The resulting cells were cultivated in LB medium with the additions of 100 μg ml^-1^ of ampicillin and 40 μg ml^-1^ of chloromycetin. When the cells reached an optical density at 600 nm of 0.6 to 0.8, the recombinant protein was induced by adding 1 mM isopropyl β-d-1-thiogalactopyranoside (IPTG). After 12 hours of incubation at 16°C, the cells were harvested and washed twice with PBS (pH 7.0), and resuspended in HisTrap buffer A (20 mM PBS, 10% glycerol, and 0.1 mM PMSF). After lysis by sonication and centrifugation twice at 30,000 × *g* for 20 minutes at 4°C, the supernatant was applied to a Ni-NTA HisTrap affinity column (GE Healthcare, Uppsala, Sweden) by using an AKTA Prime System (GE Healthcare). After washing with wash buffer B (20 mM PBS, 50 mM NaCl, 20 mM imidazole, pH 7.0), the eluted protein was collected and desalted. It was then loaded again on the Ni-NTA HisTrap affinity column as described above. Further purification was obtained by gel filtration on a Superdex 200 column (GE Healthcare) by using the AKTA Prime System. The eluted protein were collected, desalted, and concentrated by ultrafiltration on Vivaspin 20 (molecular weight cut-off, 50,000; Sartorius Stedim Biotech GmbH, Goettingen, Germany). The resulting protein fractions were analyzed by 12% SDS-PAGE and starch-containing PAGE as described by Dong *et al*. [12].

### Biochemical properties of FSA

The enzymatic activity of the purified FSA was determined by measuring the amount of reducing sugar released during the enzymatic hydrolysis of 5 g l^-1^ of soluble starch in 50 mM PBS (pH 6.0) at 50°C for 15 minutes, unless otherwise stated. Reducing sugar was measured by a modified dinitrosalicylic acid method [58]. One unit of α-amylase activity was defined as the amount of enzyme that released 1 μmol of reducing sugar as glucose per minute under the assay conditions [59].

For the determination of the optimum pH for the activity, the following buffers were used for the different pH ranges: PBS, pH 3.0 to 8.0; Tris-HCl, pH 8.0 to 9.0; glycine-NaOH, pH 9.0 to 10.0; and NaHCO_3_-NaOH, pH 10.0 to 11.0. Stability under different pH conditions was tested by preincubating the enzyme at 25°C in different pH solutions for 3 hours, and residual activity was assayed as described above. The optimal temperature range for the activity of FSA was assayed between 25°C to 80°C by using soluble starch. To determine the thermostability, the enzyme was incubated at the tested temperatures for 30 minutes before the activity measurement. In addition, FSA was incubated at 50°C in Na_2_HPO_4_ citric acid buffer (pH 6.0) in the presence or absence of 1 mM Ca^2+^. After different time intervals of incubation, the residual activities were measured under the standard assay condition.

The substrate specificity of the α-amylase was studied in 50 mM PBS (pH 6.0) at 50°C for 15 minutes. The final concentration of various substrates was 1% (w/v). The effects of various metal ions such as K^+^, Cu^2+^, Co^2+^, Zn^2+^, Ni^2+^, Cd^2+^, Ca^2+^, Mn^2+^, Mg^2+^, Ba^2+^, and EDTA were tested under the standard assay procedures, with the addition of each ion at a concentration of 5 mM. The kinetic parameters toward soluble starch were tested at 50°C in PBS buffer (pH 6.0).

### Site-directed mutagenesis

Based on the multiple alignments, Ser450 and Lys415 were replaced by cysteines respectively, using the MutanBEST kit (TaKaRa Biotechnology, Dalian, China). The mutated protein (FSAΔSK) was generated by using the primers 1f (5′-CGGGATCCGGATGACAATAATAATTATAC-3′) and 1r (5′- GACGTCGACTTACTCTC*CGC*AAACCGACC-3′) for the S450C mutagenesis, and 2f (5′-CTGGAAAGAGAAA*TGC*CTCG-3′) and 2r (5′-TTGGATAAGACGGTTCTTTG-3′) for the K415C mutagenesis. Italics indicates the amino acid sequence of mutated sites. The procedure for mutation was conducted according to the manufacturer’s instructions (MutanBEST, TaKaRa Biotechnology) and the mutants were sequenced for verification. The resulting proteins were expressed and purified as the procedures described for FSA. The purified protein was used to determine the temperature range, thermostability, and Ca^2+^ requirement for thermostability, using the methods used for FSA. Similarly, to determine the thermostability of the mutant enzyme, it was incubated at 50°C in the presence or absence of 1 mM Ca^2+^. Samples were withdrawn at various incubation periods and tested for residual α-amylase activity at 55°C under standard assay conditions.

## Abbreviations

BGI: Beijing Genomics Institute; BLA: *Bacillus licheniformis* α-amylase; CAZy: Carbohydrate-Active Enzymes; EDTA: Ethylenediaminetetraacetic acid; FLAJO: *Flavobacterium johnsoniae* α-amylase; FSA: *Flavobacteriaceae Sinomicrobium* α-amylase; GH13: Glycosyl hydrolase family 13; IPTG: Isopropyl β-D-1-thiogalactopyranoside; LB: Luria-Bertani; ORF: Open reading frame; PBS: Phosphate-buffered saline; PCR: Polymerase chain reaction; PGAAP: Prokaryotic Genome Automatic Annotation Pipeline; PMSF: Phenylmethylsulfonyl fluoride; PWA: *Pyrococcus woesei* α-amylase; RAST: Rapid annotations using subsystems technology.

## Competing interests

The authors declare that they have no competing interests.

## Authors’ contributions

CL carried out the characterization, site-directed mutagenesis, and drafted the manuscript. MD carried out the clone and expression of FSA. BC carried out the enzyme purification. CY directed the overall study and revised the manuscript. LW helped to analyze the sequence and structure. XL helped to purify and characterize FSA. CM and PX helped to revise the manuscript. All authors read and approved the final manuscript.
